# Longitudinal study of health, disease and access to care in rural Victoria: the Crossroads-II study: methods

**DOI:** 10.1186/s12889-018-5511-9

**Published:** 2018-05-30

**Authors:** Kristen M. Glenister, Lisa Bourke, Leslie Bolitho, Sian Wright, Stuart Roberts, William Kemp, Leigh Rhode, Ravi Bhat, Sönke Tremper, Dianna J. Magliano, Mike Morgan, Rodrigo Mariño, William Adam, David Simmons

**Affiliations:** 1 Department of Rural Health, University of Melbourne, Docker Street, Wangaratta, VIC Australia; 2 Department of Rural Health, University of Melbourne, 49 Graham Street, Shepparton, VIC Australia; 3 Gastroenterology, Alfred Health, Commercial Road, Prahran, VIC Australia; 4 Gateway Health, 155 High St, Wodonga, VIC Australia; 5 University of Melbourne, Shepparton Medical Centre, Graham Street, Shepparton, VIC Australia; 6 Baker Heart and Diabetes Institute, 75 Commercial Road, Prahran, VIC Australia; 7 Melbourne Dental School, University of Melbourne, Parkville, VIC Australia; 8 Western Sydney University, Locked Bag, Penrith, NSW 1797 Australia

**Keywords:** Rural health, Access, Diabetes, Liver fibrosis, Oral health, Chronic disease, Undiagnosed, Undermanaged, Mental health

## Abstract

**Background:**

High quality, contemporary data regarding patterns of chronic disease is essential for planning by health services, policy makers and local governments, but surprisingly scarce, including in rural Australia. This dearth of data occurs despite the recognition that rural Australians live with high rates of ill health, poor health behaviours and restricted access to health services. Crossroads-II is set in the Goulburn Valley, a rural region of Victoria, Australia 100–300 km north of metropolitan Melbourne. It is primarily an irrigated agricultural area.

The aim of the study is to identify changes in the prevalence of key chronic health conditions including the extent of undiagnosed and undermanaged disease, and association with access to care, over a 15 year period.

**Methods/design:**

This study is a 15 year follow up from the 2000–2003 Crossroads-I study (2376 households participated). Crossroads-II includes a similar face to face household survey of 3600 randomly selected households across four towns of sizes 6300 to 49,800 (50% sampled in the larger town with the remainder sampled equally from the three smaller towns). Self-reported health, health behaviour and health service usage information is verified and supplemented in a nested sub-study of 900 randomly selected adult participants in ‘clinics’ involving a range of additional questionnaires and biophysical measurements. The study is expected to run from October 2016 to December 2018.

**Discussion:**

Besides providing epidemiological and health service utilisation information relating to different diseases and their risk factors in towns of different sizes, the results will be used to develop a composite measure of health service access. The importance of access to health services will be investigated by assessing the correlation of this measure with rates of undiagnosed and undermanaged disease at the mesh block level. Results will be shared with partner organisations to inform service planning and interventions to improve health outcomes for local people.

## Background

High quality, contemporary, locally specific health data are essential to understanding the specific healthcare needs of rural populations. However, these data can be difficult to obtain for rural health services and rural local governments. Rural populations often experience a higher burden of chronic disease due to a culmination of older age profiles, lower incomes and levels of education, poor health behaviours, restricted access to care and multiple risk factors [1]. Rates of diabetes and cardiovascular disease [2] also remain higher in rural areas [3].

The Crossroads-I study was conducted in the Goulburn Valley (GV) region of Victoria, Australia between 2000 and 2003 and was initiated in response to poor health outcomes and health workforce shortages locally. The study assessed the prevalence of chronic disease and access to health care. Crossroads-I is one of the cohorts of the Australian and New Zealand Diabetes and Cancer Collaborative [4, 5]. The study was designed as a baseline study for future comparative epidemiological studies and remains one of the largest and most broadly focussed studies of rural health in Australia.

Since the original study, initiatives to address rural workforce shortages have succeeded in increasing availability of primary care in some areas of rural Australia [6], and availability of GPs is now reportedly equivalent to the state average in some areas of the Goulburn Valley [7]. Despite this, the Standardised mortality rate (SMR) remains higher than the state average in the region, as does hospitalisation and emergency department utilisation [7]. Thus, it is timely to repeat the original Crossroads-I study.

The aim of Crossroads-II is to identify changes in the prevalence of key chronic health conditions, including undiagnosed and undermanaged disease, and any association with variation in access to care since the original Crossroads-I study.

### Background to crossroads-I

Crossroads-I examined the prevalence of a range of health conditions and health service utilisation in a rural setting, and was designed to be longitudinal. Crossroads included 2376 households and 6316 participants [8]. A sub-sample (1454) were randomly selected to attend study ‘clinics’ to measure a range of biochemical, anthropometric and functional parameters [8]. Participants were selected from the main towns of the 6 shires surrounding the larger regional centre. Crossroads-I focussed on several key chronic diseases (diabetes [8], self-reported oral health [9], mental health, anaemia [10], asthma, chronic obstructive pulmonary disease [11], cardiovascular disease [12] as well as hypertension, other metabolic syndrome components [5], liver disease [5] and obesity [13]). Crossroads-I study remains one of the largest, most broadly focussed studies of health and access to health services among rural Australian adults, and complements other rural population health studies including the Busselton Health and Healthy Ageing studies (currently focussed on ‘Baby Boomers’) [14], the Blue Mountains studies (eye health and hearing loss) [15], the Dubbo study of the elderly [16] and the Australian Women’s longitudinal health studies [17]). The health of the rural Crossroads-I cohort has been compared to the health of other cohorts including AusDiab [18] and is one of the 18 cohorts of the Australian and New Zealand Diabetes and Cancer Collaboration [19].

### Why are studies like Crossroads necessary?

Limited data relating to the prevalence of key chronic conditions, health behaviours and measures of health service utilisation in the Goulburn Valley are available, but access to comprehensive, contemporary data at a level of less than statistical area 3 (SA3, representing areas with populations of 30,000–130,000 [20]) is difficult for the majority of chronic conditions.

This study was designed as a partnership between a Rural Health research team, local health services and local governments to provide high quality, contemporary, locally specific data on the health of their clients and their access to health services. These local organisations will be able to use the data in service planning, to determine service leakage, estimate unmet demand and to inform targeted interventions. The study will examine the factors associated with poorer than expected health outcomes in the region, despite increased availability of GPs in parts of the region.

### Contribution to field

**Fig. 1:**
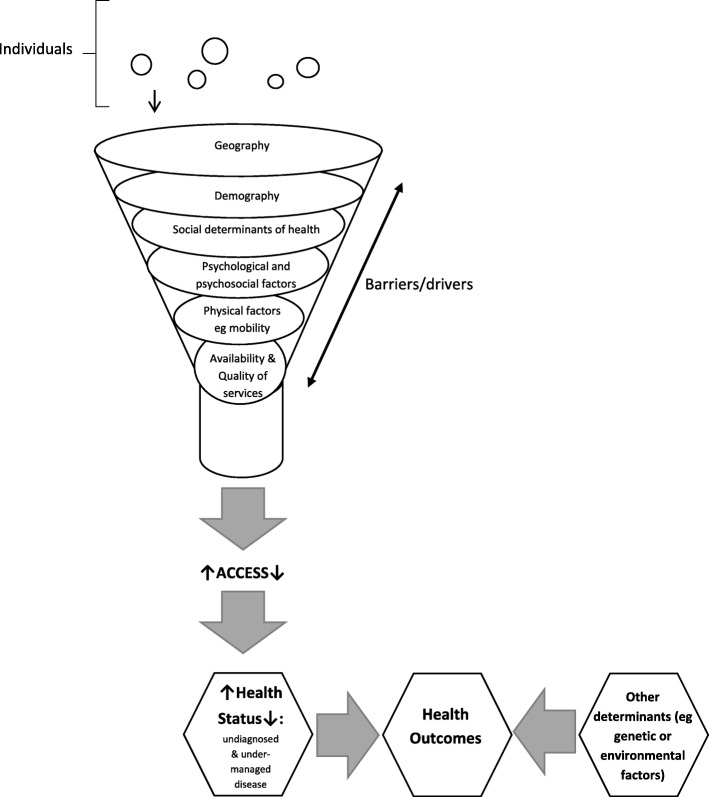
Formulation of a composite measure of access

Crossroads II will compare detailed, comprehensive population health status information between a regional centre and surrounding smaller towns, at a local level, in a rural Australian setting. The study will provide information regarding changes in health, disease and health behaviours by comparing data from two time points, 15 years apart, within the same region. The study will use data obtained at a clinic to validate self-reported health information provided by participants. A unique aspect of the study is the inclusion of a comprehensive oral health assessment. The study will formulate a composite measure of access to a range of health services and examine the impact of limitations in access on undiagnosed and/or undermanaged disease at a mesh block level (representing the smallest statistical area used by the Australian Bureau of Statistics of a maximum of 30–60 dwellings), as summarised in Fig. 1.

**Fig. 2:**
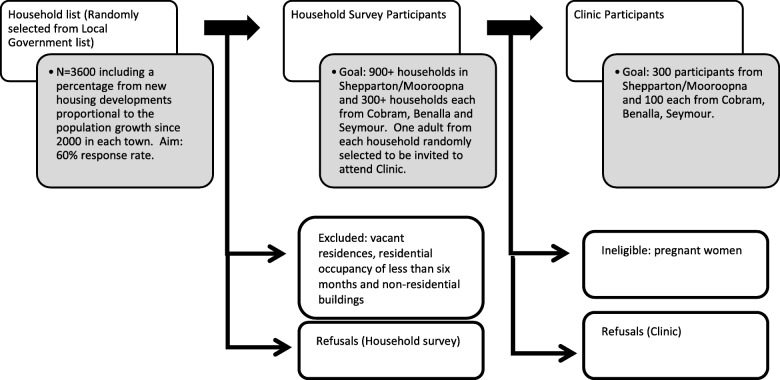
Household and clinic participant selection process

## Methods & design

### Specific aims of Crossroads-II

1. To compare the rates of self-reported, undiagnosed and undermanaged chronic disease including mental health conditions, between
  - the regional centre (Shepparton) and 3 smaller shire centres (Benalla, Cobram, Seymour) and
  - 2000–2003 baseline and 2016–2018 data.
2. To compare the self-reported waiting times and other barriers to accessing primary care, community services, oral health care, allied health, mental health and specialist care between (i) the regional centre and 3 shire centres and (ii) 2001–2003 baseline data and 2016–2018 data.
3. To develop a composite measure of healthcare access, according to the type of health service, based upon waiting times and self-reported barriers to access.
4. To correlate the composite measure of healthcare access to care with under-diagnosis and under-management of chronic disease.

### Design

This study is a repeat, cross sectional, population health study with broad focus on health, disease and access to health services. Both Crossroads-I and Crossroads-II were designed to include a household survey of randomly selected households (conducted face-to-face) to collect self-reported health, disease, health behaviour and health service usage information, with verification/supplementation of this information at the ‘clinic’. The clinic is designed as a nested study within the household survey.

### Sample size calculation

The power calculations described below are 2 tailed, alpha 0.05 with power 0.8unless otherwise stated.

#### Undiagnosed and/or undermanaged disease

Crossroads-I provided an estimate that 15% of the GV population had undermanaged and/or undiagnosed disease (Crossroads Undiagnosed Disease study, unpublished data). With 300 clinic participants from the regional centre and 300 clinic participants from shire centres, it is estimated that a 7.2% absolute difference will be able to be detected between the two groups. In addition, a 5% absolute difference is expected to be detected between the baseline study and the current study (600 vs 900 participants).

#### Access

A minimum of 1200+ household survey participants in the regional centre and 1200+ in the shire centres would enable the detection a difference of 0.33 days waiting time for health services (based on mean of 2 ± 4 days). In addition, a difference of 0.25 days waiting time between the baseline study and this study will be detectable for each health service and type of treatment provided (eg. Emergency or elective surgery).

#### Composite measure of access and undiagnosed/undermanaged disease

The correlation between access and rates of undiagnosed and/or undermanaged disease in a given area (defined by mesh block) will be calculated. 100 mesh blocks allow detection of *r* = 0.276; (24 people/mesh block from the household survey and 6 from the ‘clinic’).

### Setting

The setting for the study is the Goulburn Valley region of Victoria, Australia to allow comparison with Crossroads-I. The Goulburn Valley is located 100–300 km from the state capital of Melbourne and is a region known for irrigated agriculture. The region was chosen for Crossroads-I due to restricted access to health services and excess poor health outcomes at the time (2000–2003). For pragmatic reasons, only three (Benalla, Cobram and Seymour) of the six shire capitals from Crossroads-I will be included in this study, in addition to the regional centre of Shepparton/Mooroopna. These 3 towns were selected because of their geographical locations east, north and south of the regional centre, and their juxtaposition with other health services (another regional hospital, none, metropolitan services).

### Characteristics of participants

#### Household selection

The households included in Crossroads-I study were randomly selected from residential address lists from local government organisations in the Goulburn Valley. In Crossroads-II 3600 households will be randomly selected from the same address lists plus a proportion of households from housing developments that have been built since 2000 (using local government lists), with a target of at least 1800 participating households. The proportion of houses from new housing developments will reflect the population growth experienced by that town. If an address is confirmed to be vacant or is no longer a residential address it will be removed from the household list and replaced by another randomly selected address. Data collection commenced in October 2016. The household response rate for Crossroads-I study was 70.3% [8]. The target response rate for Crossroads-II nested study will be 60% with a minimum of 900 houses participating. Where possible, a census of household residents will be conducted for households that decline to participate.

#### Household survey

The household survey will adopt a similar approach to the Crossroads-I study, and is outlined in Fig. 2. All residents of the household aged 16 years or older who are able to provide informed consent and who have lived in the house for a minimum of 6 months will be eligible to participate. Adults will be invited to answer questions on behalf of their children aged < 16 years. The survey will be conducted face to face using iPads, with study data collected and managed using REDCap electronic data capture tools hosted at the University of Melbourne [21]. REDCap (Research Electronic Data Capture, Vanderbilt University, United States) is a secure, web-based application designed to support data capture for research studies, providing: 1) an intuitive interface for validated data entry; 2) audit trails for tracking data manipulation and export procedures; 3) automated export procedures for seamless data downloads to common statistical packages; and 4) procedures for importing data from external sources. Advertising in local newspapers, local television and presentations to local service groups will be undertaken to promote the study, along with postcards delivered to the selected households. These postcards have been translated into the four most common languages in the region other than English (namely Italian, Arabic, Persian/Dari, Turkish). People who speak a language other than English are encouraged to participate with the assistance of a family member or friend. Households are visited multiple times until it is evident that the house is unoccupied or each of the adults agree or refuse to participate.

### Clinic participants

One adult participant (≥18 years) from each participating household will be randomly selected to attend the clinic. Pregnant women will be excluded from attending the clinic. If a participant requires transport to and from the clinic, a taxi voucher is provided. If a person fails to attend the clinic, telephone contact is made to reschedule up to three times.

### Measures

#### Oral glucose tolerance test (OGTT)

All participants without diabetes will attend the clinic between 7.00 am and 10.10 am after fasting for a minimum of 8 h. These participants will have baseline blood samples taken prior to consuming the glucose solution (300 mL) containing 75 g glucose within 5 min. If a participant vomits, the test will be discontinued. Participants with known diabetes will not undergo oral glucose tolerance testing, but will have fasting blood samples taken for all other routine analytes. Participants with diabetes unable to fast (eg insulin treated with a risk of hypoglycaemia) will be able to attend non-fasting.

### General blood tests

Blood will be taken from participants by a qualified phlebotomist for the following analyses. The total volume of blood drawn will be less than 18 mL (Monovette®, Sarstedt). Blood samples will be transported in an insulated vessel and stored at room temperature prior to processing. On occasion, a data logger will be used to verify transport temperature. Blood samples will be subject to full blood examination (FBE) within the same day. Serum samples will be processed within 30 min of collection (3220 g, 10 min, 15-22 °C) and refrigerated prior to testing. All pathology testing will be completed by a centralised, registered pathology laboratory, unless otherwise specified.

**Table:** 

Test	Analyte	Undiagnosed disease threshold	Collection tube
Glucose tolerance	Baseline glucose and 2 h post glucose load	Fasting 6.1–6.9 mmol/l impaired fasting glucose 2 h 7.8-11 mmol/L impaired glucose tolerance Fasting ≥7.0 mmol/l and/or 2 h ≥ 11.1 mmol/L diabetes [22]	2.6 mL fluoride oxalate tube
Diabetes management	HbA1c	6.5% [23]	2.6 mL EDTA tube
	Insulin	> 9.0 microIU/mL predictive of pre-diabetes [24]	4.7 mL serum tube (1 mL serum aliquot to be stored at -72 °C for later analysis)
Anaemia	FBE	Haemoglobin < 130 g/L (males), < 120 g/L (Females) [25]	2.6 mL EDTA tube
Kidney function	U + E, creatinine	Urine: Albumin/Creatinine ratio: ≥2.5 (Males), ≥3.5 (Females) Plasma: Creatinine 110 μmol/L (male), 90 μmol/L (female) [26] eGFR: < 90 ml/min/1.73 m (http://www.racgp.org.au/your-practice/guidelines/redbook/8-prevention-of-vascular-and-metabolic-disease/83-cholesterol-and-other-lipids/) Sodium: outside of 134–145 mEq/L (http://www.racgp.org.au/your-practice/guidelines/redbook/8-prevention-of-vascular-and-metabolic-disease/83-cholesterol-and-other-lipids/) Potassium: outside of 3.5-5 mmol/L (http://www.racgp.org.au/your-practice/guidelines/redbook/8-prevention-of-vascular-and-metabolic-disease/83-cholesterol-and-other-lipids/) Urea: outside 3-8 mmol/L (http://www.racgp.org.au/your-practice/guidelines/redbook/8-prevention-of-vascular-and-metabolic-disease/83-cholesterol-and-other-lipids/)	4.7 mL gel tube
LFT	Gamma glutamyl transferase (GGT), alanine transaminase (ALT), aspartate transaminase (AST).	GGT: ≥ 60 U/L [27] ALT: ≥30 U/L (males), ≥20 U/L (females) [27] AST: ≥45 U/L [27]	4.7 mL gel tube
Lipids	Cholesterol, high density lipoproteins (HDL), low density lipoproteins (LDL), triglycerides.	Cholesterol: ≥4 mmol/L HDL: < 1 mmol/L LDL: > 2 mmol/L Triglycerides: ≥2 mmol/L (http://www.racgp.org.au/your-practice/guidelines/redbook/8-prevention-of-vascular-and-metabolic-disease/83-cholesterol-and-other-lipids/)	4.7 mL gel tube
Hepatitis B and C infection or immunity	Hepatitis B surface antibody Hepatitis C antibodies	Positive test plus independent confirmatory testing.	4.7 mL gel tube

### Urine test

Urine samples will be provided by participants within 2 h of the baseline blood test using a standard urine collection container. Urine samples will be tested for creatinine and albumin to calculate the urinary albumin:creatinine ratio.

### Stored serum samples

Serum samples (2x1mL aliquots) will be stored securely for future analysis including insulin and organochlorine pesticide metabolites. Organochlorine pesticide metabolites in serum will be analysed by gas chromatography/mass spectrometry [28].

### Anthropometric measurements

Height will be measured using a Seca 213 portable stadiometer with 1 mm graduations (Seca, Hamburg, Germany). Weight will be measured in triplicate using Seca 813 electronic scales with 200 kg capacity and 100 g increments (Seca, Hamburg, Germany). Calibration will be undertaken every 6 months. Waist measurement will be assessed in triplicate using medical grade measuring tape (SLD00035, MedShop). The location of the waist will be taken as the half way point between the lowest rib and the top of the iliac crest. Measuring tape will be replaced upon any evidence of stretching. Triplicate measurements will be obtained and the average taken of the two closest measurements.

### Liver stiffness (not measured in crossroads-I)

Liver stiffness will be determined for all participants using Fibroscan® (EchoSens, Paris) technology (models 402, or 530 compact, with M- or XL-probes). The liver will be localised with a KX5100 portable ultrasound (Kaixin®, China) when available, and used to determine the skin to capsule distance. The XL-probe will be used when the skin to capsule distance exceeds 24 mm. When available the Controlled Attenuation Parameter (CAP) will be used to assess hepatic steatosis. The median LSM (kPa) from a minimum of 10 valid readings and 60% success rate will be obtained with a maximum interquartile range of 30%. A Fibroscan reading of < 7.0 kPa will be considered within normal range. FibroScan has achieved 79% sensitivity and 78% specificity to detect fibrosis when liver stiffness exceeds 7 kPa [29]. 90–95% of healthy people with no liver disease will show results of < 7.0 kPa (median 5.3 kPa) [30].

### Spirometry

Spirometry will be conducted using the Vitalograph micro 6300 handheld spirometer (Vitalograph, United Kingdom) in conjunction with disposable mouthpieces (Vitalograph Safe T way) and nose clips (Vitalograph, United Kingdom). Participants will be excluded is they have had recent eye or abdominal surgery or angina. Calibration will be undertaken after each clinic of ≤20 participants. FEV1 and FVC will be determined in triplicate for each participant (adjusted according to participant height, weight, age and sex), with FEV1 < 80% of predicted considered indicative of obstructive lung disease and FVC < 80% of predicted indicative of restrictive lung disease (Lung Foundation, 2016). A FEV1/FVC ratio of < 0.7 or > 0.8 will be considered indicative of obstructive lung disease or restrictive lung disease respectively (Lung Foundation, 2016). The accuracy of the Vitalograph micro spirometer is reported as < 3% by volume, < 10% by flow with linearity error < 3% (Vitalograph).

### Blood pressure

Blood pressure will be assessed after participants have sat resting for a minimum of 5 min. The Omron HEM-907 Intellisense digital automatic blood pressure monitor (Omron, Japan) will be used, with appropriate sized cuff (Small, Medium, Large (Omron, Japan)). Calibration will be undertaken every six months. The measurement will be taken in triplicate, with 1 min between each measurement. An average of the two closest measurements will be recorded. Readings of ≥140/90 will be considered elevated (Heart Foundation).

### Atrial fibrillation screening

Screening for atrial fibrillation (AF) will be performed using the Kardia Mobile device (AliveCor®, Belgium) and an iPad Air-2 (Apple, California). Participants with a pacemaker or electronic implantable device will be excluded. Traces unable to be allocated as either normal or AF will be assessed by a physician, and if the diagnosis remains unclear this will be assessed by a second, independent physician. The Kardia device achieves sensitivity of 90–93% and specificity 76–86% for AF [31].

### Cognitive screening(not measured in crossroads-I)

Cognitive screening will be undertaken by all participants using the Montreal Cognitive Assessment (MoCA) tool (Montreal, Canada, developed by Ziad Nasreddine in 1996) and will be administered and scored by a trained research assistant. A score of 20 or above will be considered within the normal range. MoCA detects mild cognitive impairment with 90–96% sensitivity and 87% specificity, and ability to detect Alzheimer’s dementia with a specificity of 87% [32]. It has previously been used in populations from 49 to 85 years+ [32], and is considered superior to mini-mental state examination (MMSE) for mild impairment [33].

### Audiometry

Audiology assessment will be conducted using the Madsen Zeta audiometer (Otometrics, Denmark) and ME70 12 kHz noise reduction headphones by trained research assistants. Participants who use hearing aids will remove their hearing aids prior to assessment. Calibration will be conducted on a 12 month cycle. Hearing threshold (− 10 to 130 dB) at frequencies of 250, 500, 1000, 2000, 4000, 8000 Hz will be determined. The hearing test result communicated to the participants will be the pure tone average threshold of 500, 1000 and 2000 Hz, with normal hearing defined as a threshold of < 20 dB, mild hearing loss as 20-40 dB, moderate hearing loss as 41-60 dB and severe hearing loss as > 60 dB for each ear. More comprehensive analysis of hearing results will be conducted for research purposes. The accuracy of pure tone frequencies is reported to be better than 1% (Otometrics).

### Mental health screening tools

Participants will undergo a series of self-administered mental health screening exercises, namely Short Form-36 (SF-36), Patient Health Questionnaire-9 (PHQ-9), K10 and General Health Questionnaire-12 (GHQ-12). Scoring for SF-36 will be conducted for each of the 8 domains (four domains for physical health and four domains for mental health), with scores ranging from 0 to 100 [34]. Higher SF-36 scores indicate higher health related quality of life. Scoring for PHQ-9 will indicate level of depression, with 0–4 suggesting no depression, 5–9 mild, 10–14 moderate, 15–19 moderate to severe, 20–27 severe. PHQ-9 has shown high test-retest reliability for psychiatric disorders (0.85) and high sensitivity and specificity (84 and 97% respectively) [34]. The K10 has a maximum score of 50 indicating severe distress, with the minimum score of 10 indicating no distress [35]. The GHQ-12 results will be scored as 0 = no evidence of mental ill health, 1–3 = less than optimal mental health and ≥ 4 as probable mental ill health or psychological disturbance [36].

### Additional questionnaires

Participants will self-complete a series of questionnaires related to women’s health (as applicable), diet, exercise, smoking, alcohol intake, chest pain, health knowledge and attitudes, oral health, caffeine intake, marijuana use and pesticide exposure.

### Oral health assessment (not assessed in crossroads-I)

The World Health Organisation (WHO) criteria and recommendations for oral health assessment for adults will be utilised. These criteria were selected to allow comparisons with other surveys conducted in Australia using the same methodology. This assessment will include the number of tooth surfaces with evidence of dental caries (i.e., number of decayed, missing and filled tooth surfaces). These data will be used to compute Decayed, Missing, and Filled Surfaces (DMFS) index. For collecting data on the periodontal status the Community Periodontal Index (CPI) and the position of the gingival margin in relation to the cemento-enamel-junction (“Loss of attachment”) will be measured using the WHO methodology. Loss of attachment, is regarded as an invariant destructive component associated with periodontal disease. In addition, enamel fluorosis, dental erosion, dental trauma, oral mucosal lesion condition and location, as well as dental prostheses need and possession by arch will be recorded as individual scores as described in the WHO guide.

### Data management

Household survey responses will be collected directly via iPads using REDCAP software. These data are uploaded regularly to SPSS (IBM Corp., Armonk, NY). Participants will receive their clinic results, with the option of having a copy sent to their nominated General Practitioner (GP) within 1–2 weeks of the clinic. The results sent to the participants will include pathology test results, hearing test result, spirometry result, anthropometric measurements, liver fibrosis result, blood pressure, atrial fibrillation screen result and the cognitive screening result. All clinic results will be entered into an Excel spreadsheet prior to transfer to SPSS.

### Data linkage

Information about participants’ health and health service utilisation will be compared to health information obtained from specified primary health services and hospitals with informed, written consent of participants. This process will allow validation of self-reported information. Information obtained from health services will include diagnoses, frequency of utilisation, procedures, treatments, referrals, diagnostic tests, medication and management plans.

### Statistical analysis

Data analysis will involve Chi-square tests (for discrete measures) and ANOVA (for continuous measures). Data will be described using 95% confidence intervals. Correlations will use Pearson’s r. Multivariate analyses will include logistic regression and multi-linear regression analysis. All tests will be 2-tailed with significance in the analyses set at the 5% level. Mediation analysis will be undertaken to determine the mediating role of the access composite variable with under-diagnosis and under-management using a series of linear regression models in accordance with the product of coefficients mediation method [37, 38]. All models will be adjusted for socio-demographic variables, obesity and other possible confounders.

## Discussion

Access to services is a key issue in rural areas. Terry and colleagues [39] found that access to health services varied across similar rural regions of Victoria, but the study was not designed to correlate access with health outcomes. Crossroads-II provides a unique opportunity to compare access to care currently and at the time of the original Crossroads-I study, and to correlate access to care with undiagnosed and undermanaged disease. Access to care is determined by more than geography, and both Crossroads studies are able to capture many of the social aspects of access to care by identifying rural residents’ perceptions of barriers to accessing care. Developing a new measure of access that is consumer driven will contribute to the literature in rural health where access to health services is often based on health service and population size [40, 41] rather than consumer experience. Furthermore, correlating this measure with undiagnosed and undermanaged disease will directly relate access to health outcomes in four rural communities. This provides detail about specific health outcomes and access to specific types of care in particular communities rather than an aggregation of populations in diverse rural communities.

The repeat cross-sectional aspect of the study will allow identification of changes in the health and disease profile (particularly chronic disease) of the region as well as changes in health behaviours and access to health services. Localised data, particularly detailed data on a broad range of health conditions, is uncommon in Rural Health. Therefore, this study will represent one of the most comprehensive, broadly focussed studies of the health of rural Australians. The design of this study incorporates face-to-face interviews of each member of the household, and is therefore an expensive mode of research. This study will generate a large amount of data, and therefore data quality, analysis and storage have been planned in detail.

Chronic conditions remain a growing challenge to the Australian health system [42]. Chronic disease management is now a large focus of General Practice and with an older population in rural Australia, the need to understand the prevalence of chronic disease and access to care is crucial. Understanding self-reported behaviour and matching this to clinical measures provides particular insight into the health of these residents. Furthermore, assessing chronic illness over time provides evidence of the changes in health that the health system needs to be able to cater for. The study is likely to have implications for service design and delivery in rural and regional areas.

Therefore, the results will contribute to the knowledge base in rural health and enable better understanding of the key factors influencing health and their interrelationships in rural locations. The need for this type of information prompted local health services and local governments to partner with researchers, and contribute financially to the study. Data from Crossroads-I enabled these organisations to secure funding for increased workforce and service provision. The researchers are very conscious of ensuring that each partner organisation receives the information that they require, in the most appropriate format, in a timely manner, and as such, a communication committee will be developed.

The setting of the study is the same as Crossroads-I, although for pragmatic reasons the number of smaller towns was reduced from six to three. The three smaller towns were selected for their locations north, south and east of the regional centre and their distinct health service composition. The Goulburn Valley remains home to the largest population of Indigenous Australians in regional Victoria but has experienced changes in its ethnic structure due to increased migration from non-English speaking countries and increased numbers of refugee and asylum seekers from Afghanistan, Iraq and Sudan since Crossroads-I. The impact of these changes on health, disease and access to care will be investigated in this study.

## Abbreviations

AF: Atrial fibrillation
ALT: Alanine transaminase
AST: Aspartate transaminase
CAP: Controlled Attenuation Parameter
CPI: Community Periodontal Index
DMFS: Decayed, Missing, and Filled Surfaces
eGFR: Estimated Glomerular Filtration Rate
FBE: Full blood examination
GGT: Gamma glutamyl transferase
GHQ-12: General Health questionnaire-12 item
GP: General Practitioner
GV: Goulburn Valley
HbA1c: Glycated haemoglobin
HDL: High density lipoproteins
HR-QoL: Health related quality of life
kPa: Kilo pascals
LDL: Low density lipoproteins
LFT: Liver function test
MMSE: Mini-mental state examination
MoCA: Montreal Cognitive assessment
OGTT: Oral glucose tolerance test
PHQ-9: Patient Health questionnaire-9 item
SA-3: Statistical area 3
SF-36: Short form-36 item
SMR: Standardised mortality rate
U + E: Urea and electrolytes
WHO: World Health Organisation
